# A Sensitive Hydroquinone Amperometric Sensor Based on a Novel Palladium Nanoparticle/Porous Silicon/Polypyrrole-Carbon Black Nanocomposite

**DOI:** 10.3390/bios13020178

**Published:** 2023-01-23

**Authors:** Abdullah Alrashidi, Anas M. El-Sherif, Jahir Ahmed, M. Faisal, Mabkhoot Alsaiari, Jari S. Algethami, Mohamed I. Moustafa, Abdulaziz A. M. Abahussain, Farid A. Harraz

**Affiliations:** 1 Engineering College, Northern Border University, Arar 91431, Saudi Arabia; 2 Promising Centre for Sensors and Electronic Devices (PCSED), Advanced Materials and Nano-Research Centre, Najran University, Najran 11001, Saudi Arabia; 3 Department of Chemistry, Faculty of Science and Arts, Najran University, Najran 11001, Saudi Arabia; 4 Empty Quarter Research Unit, Department of Chemistry, Faculty of Science and Arts at Sharurah, Najran University, Sharurah 68342, Saudi Arabia; 5 Department of Chemical Engineering, College of Engineering, King Saud University, Riyadh 11451, Saudi Arabia

**Keywords:** porous silicon, Pd nanoparticles, hydroquinone sensor, polypyrrole, carbon black, standard addition method

## Abstract

Exposure to hydroquinone (HQ) can cause various health hazards and negative impacts on the environment. Therefore, we developed an efficient electrochemical sensor to detect and quantify HQ based on palladium nanoparticles deposited in a porous silicon-polypyrrole-carbon black nanocomposite (Pd@PSi−PPy−C)-fabricated glassy carbon electrode. The structural and morphological characteristics of the newly fabricated Pd@PSi−PPy−C nanocomposite were investigated utilizing FESEM, TEM, EDS, XPS, XRD, and FTIR spectroscopy. The exceptionally higher sensitivity of 3.0156 μAμM^−1^ cm^−2^ and a low limit of detection (LOD) of 0.074 μM were achieved for this innovative electrochemical HQ sensor. Applying this novel modified electrode, we could detect wide-ranging HQ (1–450 μM) in neutral pH media. This newly fabricated HQ sensor showed satisfactory outcomes during the real sample investigations. During the analytical investigation, the Pd@PSi−PPy−C/GCE sensor demonstrated excellent reproducibility, repeatability, and stability. Hence, this work can be an effective method in developing a sensitive electrochemical sensor to detect harmful phenol derivatives for the green environment.

## 1. Introduction

Hydroquinone (HQ) is widely utilized in various industries including textiles, pharmaceuticals, dyes, oil refinery, cosmetics, and others [1]. Therefore, HQ certainly pollutes the environment and causes a critical hazard to human health [2,3]. Even moderate HQ contact can cause a nuisance, fatigue, haziness, vomiting, etc., [4]. Therefore, the HQ is designated as one of the common hazards in the environment by the US Environmental Protection Agency and the European Union [5]. Consequently, there is an urgent need to develop an effective technique to detect HQ from environmental samples. So far, several HQ determination methods have been reported such as the spectrophotometric method [6], fluorimetric method [7], HPLC [8], GC-MS [9], and electrochemical methods [10,11,12,13]. However, the interference effect from closely related pollutants was frequently observed in spectrophotometric detections. Furthermore, the HPLC and GC-MS require huge amounts of costly solvents, sophisticated instruments, and expert hands. Due to these various challenges and slowness, these techniques are unsuitable for on-site detection. Furthermore, fluorimetric determinations often fail to reproduce investigation results. However, the electrochemical determinations have numerous benefits such as their handy nature, and being sensitive, cost-effective, suitable for on-site detection, etc., [14,15,16,17]. Therefore, the electrochemical technique would be an effective substitute HQ determination method.

Unfortunately, HQ determination using common electrodes such as GCE, PtE, and AuE is challenging due to the poor response. Moreover, metal electrodes often suffer from overpotential. Thus, it becomes crucial to design new nanomaterials to fabricate electrodes to obtain an extraordinary electrocatalytic property towards HQ. Recently, considerable efforts have been made to determine various analytes by electrochemical techniques using chemically modified electrodes (CMEs) because of their consistency, short-response-time, cost-effectiveness, simplicity, higher sensitivity, and exclusively in-situ detection [18]. Nowadays, electrode fabrication using different types of nanoparticles of transition metal oxides or sulfides, nanocomposites, conducting polymers, etc., becomes crucial [19,20,21]. Recently, porous silicon (PSi) and PSi–based nanocomposites such as SWCNTs-PSi, PSi-mesoporous carbon, Mn_2_O_3_@PSi, Ag@PSi-PANI, etc., have been used in the determination of pollutants because of their cost-effectiveness, non-toxic nature, and over-accessibility [22,23,24]. Lately, several nanostructured material-based electrochemical sensors for HQ detection have been reported [15,25]. In addition, conducting polymers such as polypyrrole, polyaniline, and polythiophene have been widely utilized as electrode modifiers because of their consistency as the host material [16,26,27]. Recently, researchers showed huge interest in electrode fabrication utilizing polymers and surfactants because of their amazing electrochemical properties [28,29]. Currently, researchers have fabricated electrodes using hybrid materials containing noble metals, silicon, carbon, metal oxides, etc., with different conducting polymers to obtain the improved electrochemical activities of hybrid nanomaterials [30,31]. Such hybrid materials are appropriate for many applications such as organo-electronic, sensing, photocatalysis, energy-storage device, supercapacitor, solar cell, bioengineering applications, etc., [32,33,34]. Additionally, polypyrrole (PPy)-doped carbon black (C) with PSi and PdNPs hybrid nanomaterial was never used in an electrochemical-sensing application. Therefore, herein, we devoted our efforts to design and develop an effective HQ sensor by utilizing the Pd@PSi−PPy−C/GCE. 

## 2. Materials and Methods

### 2.1. Materials

Powdered silicon (~40 µm), NaH_2_PO_4_, Na_2_HPO_4_, HF, HNO_3_, palladium chloride, polypyrrole-doped carbon black, and hydroquinone were purchased from Sigma Aldrich and used as received. We utilized double-distilled water for preparing all the solutions. The XPS for the Pd@PSi−PPy−C was achieved utilizing the MgKα spectrometer (JEOL, JPS 9200) under the following conditions: pass energy = 50 eV (wide-scan) and 30 eV (narrow-scan), voltage = 10 kV, and current = 20 mA. XRD spectra were recorded using the PANalytical X-ray diffractometer using Cu Kα_1/2_, λα_1_ = 154.060 p.m., λα_2_ = 154.439 p.m. radiation. A Perkin Elmer 100 spectrometer was used to record the FTIR spectra from the PSi and Pd@PSi−PPy−C nanocomposite. FE-SEM investigations were performed using an FE-scanning electron microanalyzer (JEOL-6300F, 5 kV). The elemental analysis of the as-grown Pd@PSi−PPy−C was performed by EDS (JEOL, Japan). TEM micrographs were taken at 200 kV using a JEOL JEM-2100F-UHR field emission instrument fitted out with a Gatan GIF 2001 energy filter and 1 k-CCD camera. Electrochemical investigations were performed utilizing a Zahner Zennium potentiostat (German).

### 2.2. Synthesis of the PSi, PSi−PPy-C, and Pd@PSi−PPy-C Nanocomposite

First, 2.0 g of powdered silicon was disseminated in 20 mL 48% HF and 80 mL distilled water. Then, 10 mL 70% HNO_3_ was added to the beaker dropwise under mild stirring at ambient conditions [35]. The production of nitrogen dioxide vapor indicated the end of stain etching method. We collected the PSi nanoparticles (NPs) by decantation followed by washing 2–3 times using distilled water. Finally, we dried the PSiNPs at 65 °C for 8 h. The following reaction occurred during the stain etching method:

3Si + 4HNO_3_ + 18HF → 3H_2_SiF_6_ + 4NO + 8H_2_O

We used a facile sonication method to synthesize the PSi−PPy−C nanocomposite containing 5 wt% PPy−C polymer. For this, 0.4 g PSiNPs and 0.02 g PPy−C were carefully mixed and then dispersed in 80 mL distilled water with a mild sonication for ~30 min. Lastly, the PSi−PPy−C nanocomposite was collected and dried at 60 °C.

Later, we deposited 1% PdNPs onto the PSi-PPy-C nanocomposite utilizing a photo-deposition method. Herein, 0.2 g PSi-PPy-C was disseminated in 1% methanol solution (*v*/*v*) and stirred for 5 min. Then, 500 µL of a palladium solution comprising 0.004 g palladium was added dropwise to this 1% methanol solution. Finally, we irradiated light for 24 hrs in mildly stirred conditions using a UV source from a Philips Hg lamp (illumination intensity at 350 nm: 2.0 mWcm^−2^). We collected this 1%Pd@PSi−PPy−C nanocomposite via decantation and dried it at 60 °C. This as-grown 1% Pd@PSi−PPy−C is denoted as Pd@PSi−PPy−C in this work.

### 2.3. Modification of Glassy Carbon Working Electrode Using Pd@PSi−PPy−C Nanocomposite

Glassy carbon electrodes (GCEs) were cleaned, respectively, utilizing 1 µm diamond followed by 0.05 µm alumina. Later, GCEs were modified with the Pd@PSi–Ppy–C nanocomposite utilizing the Nafion solution. In the fabrication process, a 3.0 mg Pd@PSi–PPy–C was homogeneously mixed in 0.05 mL Nafion-0.45 mL propan-2-ol mixture and then optimized 1.5 µL suspension was cautiously transferred to clean, polished GCEs and dried at 60 °C for 20 min. Such modified GCEs are denoted as Pd@PSi−PPy−C/GCE. For the control experiments, PSi/GCE, and PSi-PPy-C/GCE were also fabricated by similar procedures. A standard 3-electrode electrochemical cell was used where a Pd@PSi−PPy−C/GCE, Ag/AgCl, and a platinum spiral were utilized as a working electrode, a reference electrode, and a counter electrode, respectively. The electrochemical investigations of HQ (1–700 µM) were carried out using cyclic voltammetry (CV), electrochemical impedance spectroscopy (EIS), and amperometry at room temperature utilizing 0.1 M PBS of pH 7.0.

## 3. Results and Discussion

### 3.1. Characterization of the Pd@PSi−PPy−C Nanocomposite 

**Figure 1:**
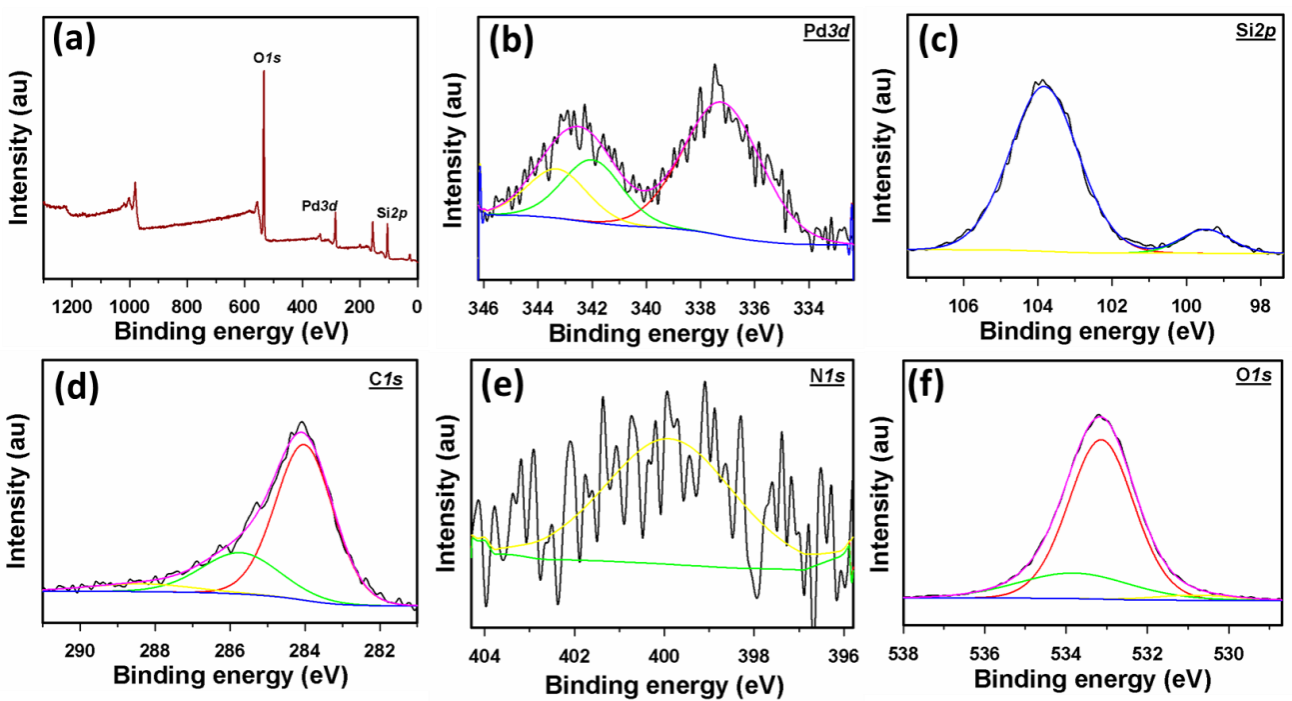
(**a**) Survey XPS spectrum of Pd@PSi−PPy−C nanocomposite. (**b**) Fine scan XPS spectrum of Pd-3d, (**c**) Si-2p, (**d**) C-1s, (**e**) N-1s, and (**f**) O-1s orbitals of Pd@PSi−PPy−C nanocomposite.

XPS was used to study the purity and structure of Pd@PSi−PPy−C. From the XPS study presented in Figure 1a–f, it is clear that this Pd@PSi−PPy−C nanocomposite was composed of Si, Pd, C, N, and O atoms only. The Pd-3d spectrum shows two well-resolved peaks (Figure 1b) that appeared at 337.2 and 342.1 eV and that can be correlated to Pd3d_5/2_ and Sb3d_3/2_, respectively. These are also consistent with the binding energies of Pd3d [36]. In the fine scan of the Si-2p XPS spectrum (Figure 1c), the peaks at 99.5 and 103.2 eV were correlated to Si2p_3/2_ and Si2p_1/2_, respectively [37]. In the deconvoluted C-1s spectrum (Figure 1d), three peaks appeared at 284.1, 285.4, and 288.1 eV, and of these, the peaks at 284.1 and 285.4 eV could be consigned to C–C and C-O-H bonds, respectively [38,39], and the remaining peak at 288.1 eV was related to COOH [40]. A previous report recommended that the C-1s peak appearing at 285.4 eV was also correlated to the C–N bonds of PPy [41]. A deconvolution plot of the N-1s spectrum (Figure 1e) displayed a peak at 399.9 eV that was related to the C–N bond of PPy moiety [42]. Figure 1f shows two peaks that appeared at 533.1 and 533.7 eV from the deconvoluted O1s spectrum that could be correlated to Si-O and C-O bonds, respectively [43].

**Figure 2:**
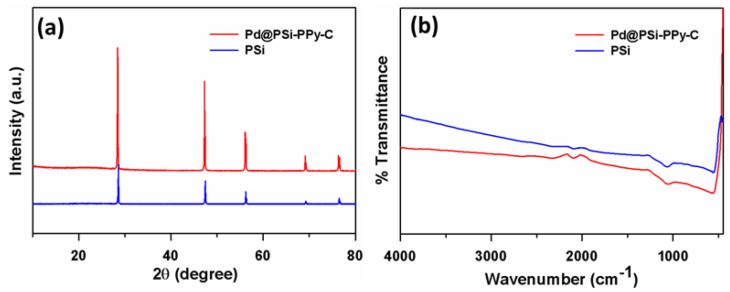
Structural and optical evaluation of Pd@PSi–PPy–C nanocomposite: (**a**) XRD patterns and (**b**) FTIR spectra.

In the XRD patterns (Figure 2a), the diffraction bands that appeared at 2θ = 28.3°, 47.3°, 56.0°, 69.1°, and 76.3° were related to (2 2 0), (3 1 1), (4 0 0), (3 3 1), and (4 2 2) lattice planes for Si (JCPDS # 27-1402), respectively [44]. The carbon-related peak of carbon black present in the Pd@PSi–PPy–C often appeared at 2θ = 24.3°, which was correlated to (0 0 2) plane [21], which is not properly visible in Figure 2a due to its sluggish intensity. Because of the low palladium content (1%) in Pd@PSi–PPy–C, the PdNPs peaks were not visible in the XRD patterns; however, the presence of PdNPs in the Pd@PSi–PPy–C was confirmed by XPS, EDS, SEM, and TEM. Figure 2b displays the FTIR investigation results of the Pd@PSi–PPy–C nanocomposite. Characteristic PSi NPs’ vibrational bands appearing at 2287 and 2095 cm^−1^ could be correlated to Si–H_2_ and Si–H stretching modes of vibration, respectively [45]. Distinct FTIR peaks that appeared at 1075 and 559 cm^−1^ were attributed to asymmetric Si–O stretching modes of vibrations [46,47].

**Figure 3:**
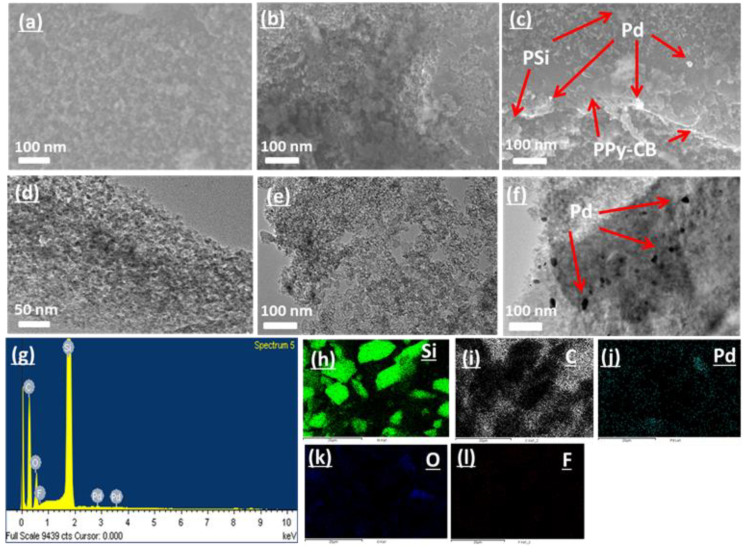
Morphological and elemental analyses: FESEM image from (**a**) PSi, (**b**) PSi−PPy−C, (**c**) Pd@PSi−PPy−C; TEM micrograph from (**d**) PSi, (**e**) PSi−PPy−C, (**f**) Pd@PSi−PPy−C; (**g**) EDS spectrum of Pd@PSi−PPy−C; (**h**–**l**) elemental mapping of Pd@PSi−PPy−C nanocomposite.

The morphology and surface structure of PSi, PSi-PPy−C, and the Pd@PSi−PPy-C nanocomposite were explored by FESEM (Figure 3a–c). The Pd@PSi−PPy−C nanocomposite consisted of PdNPs that were dispersed randomly on the porous polymeric sheet structure of PSi−PPy−C. The average diameter of PdNPs was estimated as 18 nm. TEM images in Figure 3d–f displayed a more clear morphology of PSi, PSi−PPy−C, and Pd@PSi−PPy−C, where a gathering of spherical PdNPs dispersed on sheet-like structures of PSi−PPy−C. The porous nature of the PSi and the dispersed PdNPs having an average particle size of ~19 nm was evidently labelled in the TEM micrographs. Elemental compositions of the Pd@PSi−PPy−C nanocomposite were investigated by EDS (Figure 3g) and confirmed that the Pd@PSi−PPy−C nanocomposite consisted of Pd, Si, C, F, and O only and their weight percentages were 0.97%, 50.64%, 38.47%, 0.07%, and 9.85%, respectively. Elemental mapping Figure 3h–l also confirmed the constituents of the Pd@PSi−PPy−C nanocomposite. This elemental composition of the Pd@PSi−PPy−C nanocomposite is well-matched to the above XPS and XRD results.

### 3.2. Hydroquinone Sensor Development

#### 3.2.1. Electrochemical Investigation of Pd@PSi−PPy−C/GCE

**Figure 4:**
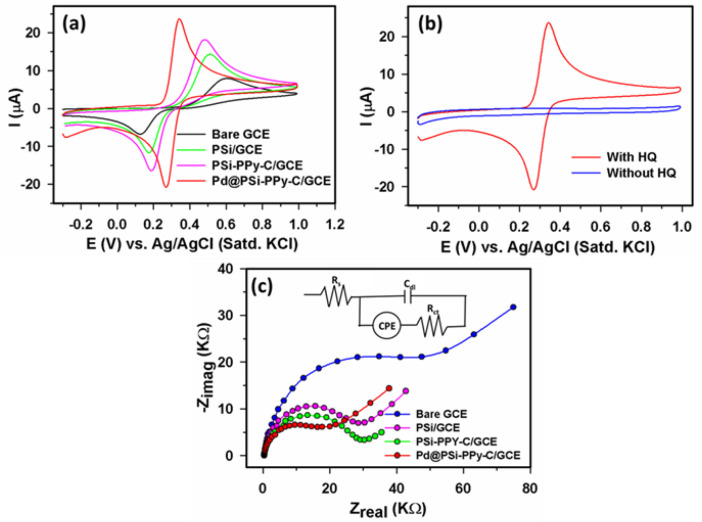
For control experiments, CVs acquired at scan rate 0.04 Vs^–1^ in 0.1 M PBS (pH 7.0): (**a**) CVs from bare GCE, PSi/GCE, PSi−PPy−C/GCE, Pd@PSi−PPy−C/GCE with 350 µM HQ; (**b**) CVs from the Pd@PSi–PPy–C/GCE with 350 µM HQ and without HQ; and (**c**) EIS Nyquist plots recorded for different electrodes in 1.0 mM [Fe(CN)_6_]^3−/4−^ in 0.1 M KCl at +0.50 V, at a signal amplitude 10 mV and a frequency ranging from 0.1 Hz to 100 KHz. The inset shows the corresponding equivalent circuit.

We explored the electrochemical activities of fabricated electrodes by cyclic voltammetry (CV) and electrochemical impedance spectroscopy (EIS). Figure 4a displays a weak CV output resulting from the bare GCE with 350 µM HQ at +0.61 V. PSi/GCE and PSi−PPy−C/GCE electrodes produced an improved CV response in the presence of 350 µM HQ at +0.52 V and +0.49 V, respectively vs. Ag/AgCl; however, the significantly improved CV result at +0.34 V was attained for Pd@PSi−PPy−C/GCE. Therefore, it was confirmed that the Pd@PSi−PPy−C/GCE electrode showed the best electrocatalytic activities during the HQ detection compared to other electrodes specified in Figure 4a. Hence, we nominated the Pd@PSi−PPy−C/GCE electrode as the HQ sensor in this work. Additionally, for the Pd@PSi−PPy−C/GCE electrode, a distinctive CV peak was achieved with 350 µM HQ, but, in the absence of HQ, no response was observed (Figure 4b) that further established the effective electrochemical properties of the Pd@PSi−PPy−C/GCE HQ sensor. Figure 4c shows the EIS Nyquist plots for the bare GCE, PSi/GCE, PSi−PPy−C/GCE, and Pd@PSi−PPy−C/GCE and the corresponding equivalent circuit is given in the inset. A low semicircular diameter obtained for the Pd@PSi−PPy−C/GCE suggested a lowered R_ct_ value (32 kΩ) of this electrode compared to the bare GCE (78 kΩ), PSi/GCE (47 kΩ), and PSi−PPy−C/GCE (39 kΩ) [48,49]. Therefore, we conclude that the Pd@PSi−PPy−C/GCE electrode exhibited enhanced electron transfer capability in comparison to other electrodes examined in Figure 4c.

**Figure 5:**
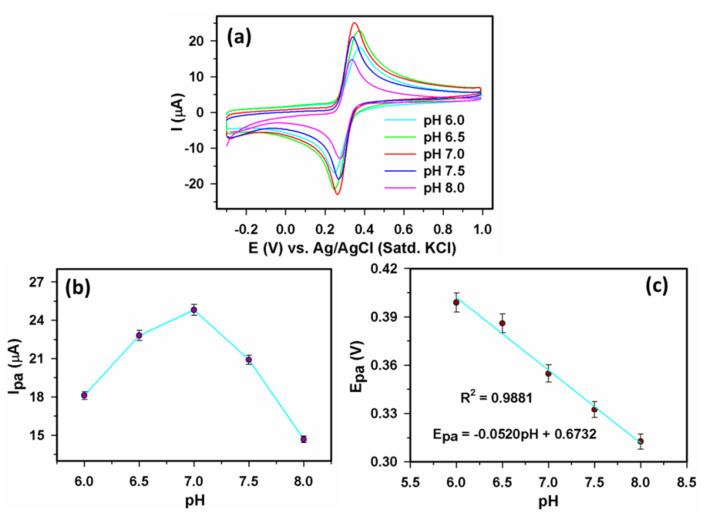
(**a**) CVs acquired for 350 µM HQ in 0.1 M PBS for varying pH (6.0–8.0) at 0.04 Vs^−1^ scan rate, (**b**) I_pa_ vs. pH, and (**c**) E_pa_ vs. pH.

To explore the electrochemical oxidation of HQ, we studied the pH effect in the range of 6.0–8.0 in the presence of 350 µM HQ. Figure 5a,b show that the I_pa_ value gradually increased for pH 6.0–7.0, whereas a decreasing trend for pH 7.0–8.0 was detected, and thus the optimal I_pa_ was observed at pH ~ 7.0 in Figure 5b. Consequently, pH 7.0 was fixed for the rest of the experiments in this work. Figure 5c displays a straight line plot of E_pa_ vs. pH having the regression Equation (1).

E_pa_(V) = 0.6732−0.0520pH     (R^2^ = 0.9881) (1)

Figure 5c shows that for the examined pH range of 6.0–8.0, a gradient −52 mV per pH unit was very close to the theoretical value −59, confirming that the transferred electron and proton numbers associated with this HQ oxidation were equivalent [16,24].

**Figure 6:**
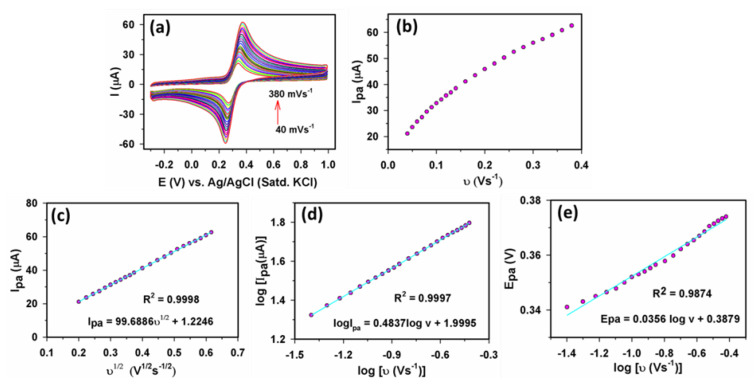
Exploration of the effect of scan rate for Pd@PSi–PPy–C/GCE electrode: (**a**) CVs for different scan rates (40–380 mVs^−1^) in the presence of 350 μM HQ (**b**) I_pa_ vs. υ, (**c**) I_pa_ vs. √υ, (**d**) log(I_pa_) vs. log(υ), and (**e**) E_pa_ vs. log(υ).

The scan rate (υ) study in Figure 6a shows the CVs of 350 µM HQ recorded for varying scan rates (40–380 mVs^−1^) utilizing the Pd@PSi−PPy−C/GCE electrode. The I_pa_ value in Figure 6a increased with increasing υ, while the E_pa_ values were only slightly shifted in the positive directions. Figure 6b exhibits the nonlinear variation of I_pa_ vs. υ indicating that the HQ oxidation did not follow a surface-controlled process [50,51]. Moreover, for υ > 0.04 Vs^−1^, a linear I_pa_ vs. υ^1/2^ curve was also obtained in Figure 6c, confirming a diffusion-controlled process [5,52,53,54] according to the following Equation (2).

I_pa_(µA) = 99.6886 υ^1/2^ (V^1/2^s^−1/2^) + 1.2246     (R^2^ = 0.9998) (2)

Again, Figure 6d displays a linear relation between log(I_pa_) and log(υ) with the following Equation (3) confirming the diffusion-controlled process [11].

log[I_pa_ (µA)] = 0.4837 log [υ (Vs^−1^)] + 1.9995    (R^2^ = 0.9997) (3)

Furthermore, in Figure 6e, another linear plot of E_pa_ vs. log(υ) was achieved with the following Equation (4).

E_pa_(V) = 0.0356 log[υ (Vs^−1^)] + 0.3879     (R^2^ = 0.9874) (4)

Figure 6a exhibits that for υ < 150 mVs^−1^, [E_pa_–E_pc_]/2 stayed nearly the same as 48.3 mV. Therefore, at the 50 mVs^−1^ scan rate, [E_pa_–E_pc_]/2 can be taken as 90.5/n_α_ mV [1], and thus the number of transferred electrons (n_α_) was calculated as 1.87 ≈ 2. Thus, we decided that the HQ oxidation at the Pd@PSi–PPy–C/GCE sensor was involved in transferring two electrons. Therefore, the scan rate and the pH studies established that the HQ oxidation at the Pd@PSi–PPy–C/GCE sensor was a combination of two-electron plus two-protons, which is in line with the literature [1]. 

#### 3.2.2. Determination of Sensor Parameters for the Pd@PSi–PPy–C/GCE Sensor

**Figure 7:**
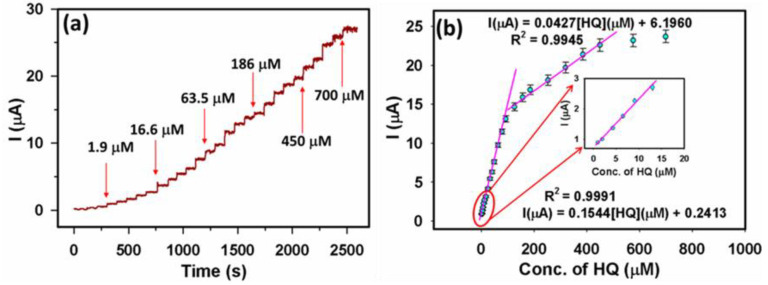
(**a**) Amperometric responses recorded at +0.35 V utilizing Pd@PSi–PPy–C/GCE sensor with HQ (1–700 µM) and (**b**) corresponding calibration plot.

We used the amperometric technique to explore the sensor performance of the Pd@PSi–PPy–C/GCE sensor. The amperometric (*i*–*t*) response was acquired at the optimized potential of +0.35 V with successive HQ additions with varying concentrations (1–700 µM). Figure 7a displays the amperometric *i-t* curve accomplished with HQ using the Pd@PSi−PPy−C/GCE assembly. Here, for each HQ addition, the current response reached ~96% of its highest current in only 6 s. Figure 7b displays two linear segments in the calibration plot: for the lower concentration region, 1–13 µM HQ, and for the higher concentration part, 13–450 µM HQ plotted based on the amperometric responses having the following Equations (5) and (6), respectively.

I(μA) = 0.1544 [HQ](µM) + 0.2413     (R^2^ = 0.9991) (5)

I(μA) = 0.0427 [HQ](µM) + 6.1960     (R^2^ = 0.9945) (6)

Thus, the linear dynamic range (LDR) for the Pd@PSi–PPy–C/GCE sensor was obtained as 1–450 µM. Furthermore, the estimated sensitivity value for the Pd@PSi−PPy−C/GCE sensor was achieved as 3.0156 µAµM^–1^cm^−2^, the LOD calculated as ~ 0.074 μM (S/N = 3), and the limit of quantification (LOQ) as 0.227 µM. For the sensitivity calculation, we used the equation sensitivity = S/A_eff_ [11], where A_eff_ denotes the surface area of the active electrode (0.0512 cm^2^) as presented in the electronic supplementary materials [55]. The LOD and LOQ were estimated using equations LOD = 3.3(S_b_/S) and LOQ = 10(S_b_/S), respectively [56,57]; herein, the S_b_ (0.0035) stands for RSD of five blank runs and S represents the slope of the calibration curve. 

In electrochemical kinetics, electrocatalytic activities depend on two factors: (i) the intensification of the current responses and (ii) the reduction of overpotential during the electrooxidation. Consequently, we tried to enhance the electrochemical property of the active electrode by fabricating the working GCEs with the active Pd@PSi−PPy−C nanocomposite. The obtained results confirmed that this Pd@PSi−PPy−C/GCE sensor effectively fulfills both of the above-mentioned factors. The above Figure 4a shows a more substantial negative shift of E_pa_ and massive I_pa_ increase from the Pd@PSi−PPy−C/GCE sensor than other electrodes employed here. Actually, we attained ~3 times the I_pa_ value than the bare GCE during the HQ oxidation at the Pd@PSi−PPy−C/GCE.

#### 3.2.3. Selectivity, Repeatability, Reproducibility, and Stability of Modified Electrodes

**Figure 8:**
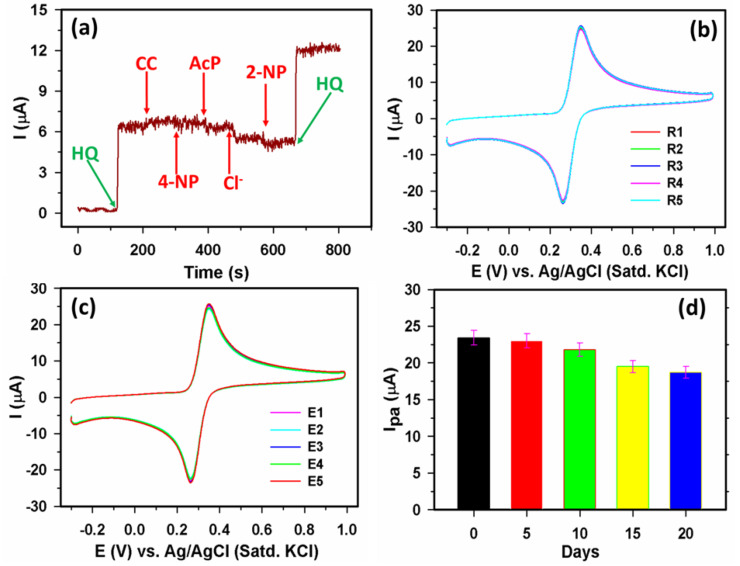
(**a**) Amperometric (*i-t*) response recorded at +0.35 V utilizing Pd@PSi−PPy−C/GCE upon successive additions of 20 µM of HQ, CC, 4-NP, AcP, Cl^−^, 2-NP, and HQ; (**b**) repeatability study; (**c**) reproducibility study; and (**d**) stability study.

For the verification of selectivity of the Pd@PSi−PPy−C/GCE sensor using common interfering chemicals such as catechol (CC), 4-nitrophenol (4-NP), 2-nitrophenol, (2-NP), 4-acetamidophenol (AcP), and Cl^−^ ions, the amperometric response was noted with 20 μM HQ with the same quantity of each interfering chemical (Figure 8a). Herein, the HQ addition produced a current response, but for the interfering chemicals, an insignificant response was observed. Thus, we confirmed the selectivity of the Pd@PSi−PPy−C/GCE sensor in HQ determination. Additionally, other sensor parameters of Pd@PSi−PPy−C/GCE were also studied utilizing CV using 350 µM HQ at the 0.04 Vs^−1^ scan rate. Figure 8b displays the repeatability study, in which the newly modified Pd@PSi−PPy−C/GCE electrode was utilized in determining 350 μM HQ. An almost-identical CV response was achieved in five runs having 3.8% RSD establishing excellent repeatability. Figure 8c shows a reproducibility investigation for the Pd@PSi−PPy−C/GCE sensor, in which five freshly fabricated Pd@PSi−PPy−C/GCE electrodes were utilized. The CV results showed a 4.2% RSD for I_pa_ variations, confirming excellent reproducibility. Following the fabrication of the Pd@PSi−PPy−C/GCE sensor, we collected CVs every fifth day in a row to test the stability and kept it at ambient conditions. A bar graph of the stability analysis is shown in Figure 8d. It reveals that after 20 days of storage at ambient conditions, the CV response remained at, or near, 85% of its initial value, and the Pd@PSi−PPy−C/GCE surface remained undamaged.

When the HQ molecule touched the Pd@PSi−PPy−C surface, an electrooxidation reaction occurred. Due to the reducing properties of HQ molecules, the electron donation from HQ to the conduction band of the Pd@PSi−PPy−C nanocomposite can occur, which ultimately enhances the Pd@PSi−PPy−C/GCE sensor’s conductivity. Thus, the CV responses are improved. The current Pd@PSi−PPy−C/GCE sensor showed an extreme sensitivity during HQ detection compared to existing HQ sensors as shown in Table 1 [4,58,59,60,61,62,63,64].

**Scheme 1:**
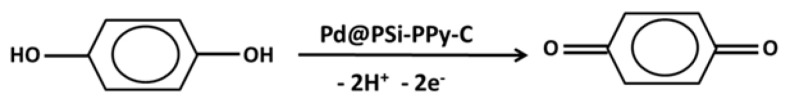
Schematic representation of electrochemical oxidation of HQ at Pd@PSi−PPy−C/GCE sensor electrode.

Therefore, it is concluded that the Pd@PSi−PPy−C nanocomposite is exceptionally efficient to oxidize HQ. The Pd@PSi−PPy−C nanocomposite provides an encouraging nano-environment in HQ detection. The PSi, PPy−C, and combined PSi−PPy−C nanocomposites are *p*-type semiconductors [24]. Hence, a combined PdNPs and PSi−PPy−C may generate metal-semiconductor (MS) junctions that provide synergistic effects [65]. A low R_ct_ value of Pd@PSi−PPy−C/GCE as achieved from the EIS spectrum (Figure 4c) further confirms the synergistic effects between PdNPs and PSi−PPy−C. Such a combination might produce an electron donor–acceptor pair resulting in a potential development at the MS junction, which may lead to a decrease in the energy barrier in the oxidation process [35]. Such a Pd@PSi−PPy−C nanocomposite–HQ interaction may be the main reason that makes the Pd@PSi−PPy−C/GCE appropriate in HQ detection. During the HQ oxidation, the dispersed PdNPs onto the PSi- PPy-C surface expedite HQ molecules’ congregation at the electrode/solution interface, and this increases the electrode sensitivity in HQ sensing [66]. The effective Pd@PSi−PPy−C nanocomposite–HQ interactions enable the Pd@PSi−PPy−C/GCE sensor appropriate for HQ determination. Furthermore, the enhanced performance of the currently developed Pd@PSi−PPy−C/GCE-based HQ sensor is also likely related to the efficient attachment of the active nanocomposite onto the GCE surface, which provides a 56% higher effective surface area than the bare, unmodified GCE, facilitating a rapid electron transfer during the electrooxidation of HQ. Scheme 1 denotes the electrooxidation of HQ at the Pd@PSi−PPy−C/GCE assembly. 

#### 3.2.4. Real Sample Investigation

To confirm the sensor electrode appropriateness, we detected HQ from spiked tap water utilizing the Pd@PSi−PPy−C/GCE sensor via a standard addition method. Herein, equal volumes of HQ solutions (varying concentrations) and tap water were mixed individually, and we recorded the CV responses in PBS utilizing the Pd@PSi−PPy−C/GCE sensor. Table 2 summarizes the results achieved, which demonstrated that the Pd@PSi−PPy−C/GCE sensor exhibited ~100% recovery of HQ. Consequently, we concluded that the Pd@PSi−PPy−C/GCE assembly is suitable, precise, and consistent for the determination of HQ from a real sample. 

**Scheme 2:**
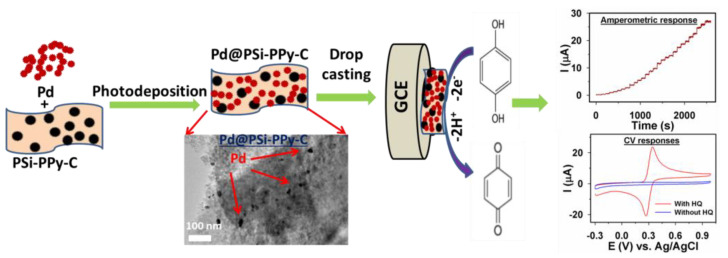
Schematic representation of nanocomposite synthesis and HQ detection at the Pd@PSi−PPy−C/GCE sensor.

Herein, during the HQ detection, the electrochemical response progressively increased with the increasing HQ concentrations. Thus, HQ became oxidized at the Pd@PSi−PPy−C/GCE sensor surface by losing two electrons to the conduction band of the Pd@PSi−PPy−C nanocomposite that originated the electrochemical response [1]. Therefore, the overall senor activity of the Pd@PSi−PPy−C/GCE can be presented as in Scheme 2. 

## 4. Conclusions

We successfully synthesized and systematically characterized a new ternary Pd@PSi−PPy−C nanocomposite via facile stain etching, sonication, and photodeposition procedures. An electrochemical hydroquinone sensor with extremely high sensitivity was designed using the Pd@PSi−PPy−C−fabricated GCE. A significantly high sensitivity value indicated the suitability of the Pd@PSi−PPy−C/GCE sensor in determining the wide-ranging HQ. Exceptional promising features of the Pd@PSi−PPy−C/GCE sensor including outstanding stability and lower LOD established the prospective of the ternary Pd@PSi−PPy−C nanocomposite during HQ detection and quantification. The reliability of this newly designed HQ sensor was confirmed utilizing spiked tap water investigations with promising analytical results. Therefore, the ternary Pd@PSi−PPy−C nanocomposite-based electrochemical sensor design method paves a new route to develop efficient electrochemical sensors to detect and quantify pollutants.

## Figures and Tables

**Table 1:** Comparison of main sensor parameters of the currently developed sensor electrode toward HQ detection by the electrochemical approach with various modified reported electrodes.

Material	Method	LDR/μM	LOD/ µM	Sensitivity/ μAμM^−1^	Ref.
Gr–COOH	ASV	0.1–40	0.04	1.390	[4]
NiO/ILs.	SWV	0.1–500	0.05	0.3425	[58]
Fe_2_O_3_/SWCNT	DPV	1–260	0.5	1.24	[59]
Au–gC_3_N_4_–MOF	DPV	0.005–5	0.001	-	[60]
Al–MOF	Amp	0.5–1500	0.067	1.4714 *	[61]
pAPBA/MWCNTs	DPV	0.5–40	0.2	-	[62]
CNCs/Zn–TPP	DPV	25–1500	0.9	0.4800 *	[63]
c–MWNTs	DPV	10–120	2.3	-	[64]
Pd@PSi–PPy–C	Amp	1–13 13–450	0.074 -	3.0156 0.8340 *	This work

* = μAμM^−1^cm^−2^, CNCs = carbon nanocoils, TPP = tetraphenylporphyrin, pAPBA = poly(3-amino-phenyl boronic acid), Gr-COOH = carboxylic acid-functionalized graphene, ILs = ionic liquids, Au-gC_3_N_4_-MOF = Zn-MOF-nitrogen doped graphite-Au NPs, ASV = anodic stripping voltammetry.

**Table 2:** HQ detection and quantification from spiked tap water using the currently developed Pd@PSi−PPy−C-modified GCE.

Real Sample	HQ Added (μM)	HQ Detected (μM)	Recovery (%)	RSD (%) (*n* = 3)
Tap water	10	9.63	96.3	3.76
	20	19.92	99.6	4.18
	30	29.68	98.9	3.85

## Data Availability

Data will be applicable upon request.

## Supplementary Materials

The following supporting information can be downloaded at: https://www.mdpi.com/article/10.3390/bios13020178/s1, Figure S1: (a) CVs recorded using the Pd@PSi−PPy−C/GCE electrode in 5 mM [Fe(CN)6]3-/4- in 0.1 M KCl (b) Corresponding Ipa vs. ʋ1/2 plots for the Pd@PSi−PPy−C/GCE electrode.
