# Supplementary material for: A Sensitive Hydroquinone Amperometric Sensor Based on a Novel Palladium Nanoparticle/Porous Silicon/Polypyrrole-Carbon Black Nanocomposite
Source: Biosensors (Basel). 2023 Jan 23;13(2):178. doi: 10.3390/bios13020178 (PMC9953257; doi:10.3390/bios13020178)
Supplement: Supplementary file 1 [file biosensors-13-00178-s001.zip › biosensors-2127438-supplementary.pdf]

Supplementary Material

Calculation of effective electrode surface area:

We recorded CV responses utilizing the Pd@PSi–PPy–C/GCE and bare GCE for varying scan rates ranging from 10 to 100 mVs^{-1} using 5 mM $[Fe(CN)_6]^{3-/4-}$ in 0.1 M KCl. Later, we plotted the CVs and I_{pa} vs. $\upsilon^{1/2}$ for Pd@PSi–PPy–C/GCE electrode **(Fig S1)** to calculate the effective surface areas using the Randles-Sevcik equation.

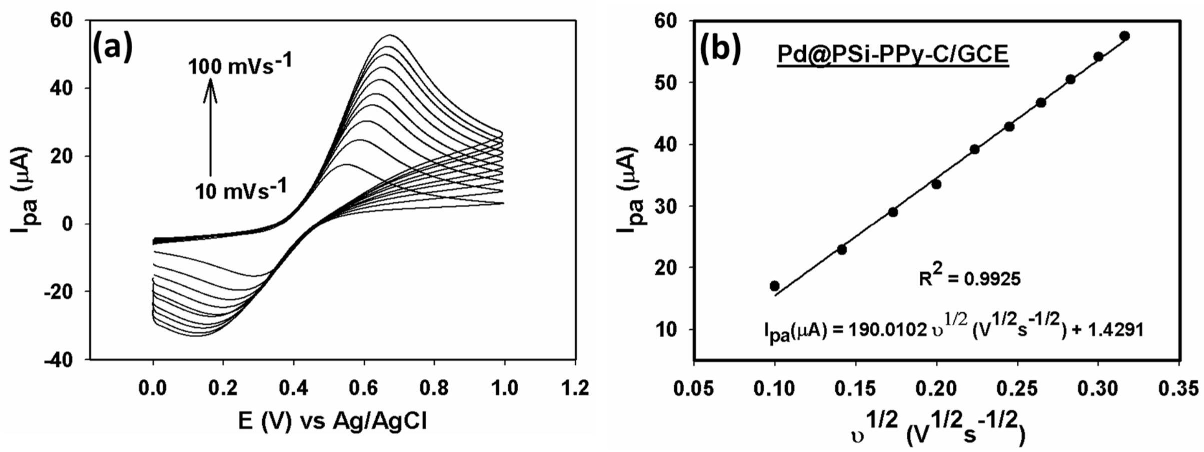

Fig. S1. (a) CVs recorded using the Pd@PSi–PPy–C/GCE electrode in 5 mM $[Fe(CN)_6]^{3-/4-}$ in 0.1 M KCl (b) Corresponding I_{pa} vs. $\upsilon^{1/2}$ plots for the Pd@PSi–PPy–C/GCE electrode

By putting the slope (1.9001×10^{-4} $AV^{-1/2}s^{1/2}$) of the i_p vs. $\upsilon^{1/2}$ plot (Fig. S1a) in Randles-Sevcik equation, $i_p = (2.69\times10^5)\ n^{3/2}\ A_{eff}\ D^{1/2}\ C_o\ \upsilon^{1/2}$ we got

$$1.9001\times10^{-4} = (2.69\times10^5)\ n^{3/2}\ A_{eff}\ D^{1/2}\ C_o$$

Now, by using the values of n =1, D = 7.6 × 10^{-6} cm^2s^{-1} is the diffusion-coefficient of $[Fe(CN)_6]^{3-/4-}$ [1–3], C_o = 5.0×10^{-6} $molcm^{-3}$ is the concentration of $[Fe(CN)_6]^{3-/4-}$, we calculated the effective surface area of the fabricated Pd@PSi-PPy-C/GCE electrode below:

$$A_{eff} = (1.9001\times10^{-4}) / [2.69\times10^5 \times 1^{3/2}\times (7.6\times10^{-6})^{1/2}\times (5.0\times10^{-6})] = 0.0512\ cm^2$$

Similarly, we have calculated the effective surface area of the bare GCE and obtained as 0.0328 cm^2.

(Those references have been cited in the main text with numbers: 24, 16, and 11 respectively)

References

1. Ahmed, J.; Faisal, M.; Harraz, F.A.; Jalalah, M.; Alsareii, S.A. Porous Silicon-Mesoporous Carbon Nanocomposite Based Electrochemical Sensor for Sensitive and Selective Detection of Ascorbic Acid in Real Samples. *J. Taiwan Inst. Chem. Eng.* **2021**, *125*, 360–371. https://doi.org/10.1016/j.jtice.2021.06.018.

2. Ahmed, J.; Faisal, M.; Jalalah, M.; Alsaiari, M.; Alsareii, S.A.; Harraz, F.A. An Efficient Amperometric Catechol Sensor Based on Novel Polypyrrole-Carbon Black Doped α-Fe2O3 Nanocomposite. *Colloids Surf. A Physicochem. Eng. Asp.* **2021**, *619*, 126469. https://doi.org/10.1016/j.colsurfa.2021.126469.

3. Ahmed, J.; Faisal, M.; Jalalah, M.; Alsareii, S.A.; Harraz, F.A. Novel Polypyrrole-Carbon Black Doped ZnO Nanocomposite for Efficient Amperometric Detection of Hydroquinone. *J. Electroanal. Chem.* **2021**, *898*, 115631. https://doi.org/10.1016/j.jelechem.2021.115631.
